# B cell abnormalities and autoantibody production in patients with partial RAG deficiency

**DOI:** 10.3389/fimmu.2023.1155380

**Published:** 2023-07-05

**Authors:** Qing Min, Krisztian Csomos, Yaxuan Li, Lulu Dong, Ziying Hu, Xin Meng, Meiping Yu, Jolan E. Walter, Ji-Yang Wang

**Affiliations:** ^1^ Department of Clinical Immunology, Children’s Hospital of Fudan University, National Children’s Medical Center, Shanghai, China; ^2^ Division of Pediatric Allergy/Immunology, University of South Florida at Johns Hopkins All Children’s Hospital, St. Petersburg, FL, United States; ^3^ Department of Immunology, School of Basic Medical Sciences, Fudan University, Shanghai, China; ^4^ Department of Microbiology and Immunology, College of Basic Medical Sciences, Zhengzhou University, Zhengzhou, China; ^5^ Division of Pediatric Allergy/Immunology, Massachusetts General Hospital for Children, Boston, MA, United States; ^6^ Shanghai Huashen Institute of Microbes and Infections, Shanghai, China

**Keywords:** RAG deficiency, B cell tolerance, BAFF, homeostatic proliferation, double negative B, CD11c^high^Tbet^+^ B, autoantibody production

## Abstract

Mutations in the recombination activating gene 1 (*RAG1*) and *RAG2* in humans are associated with a broad spectrum of clinical phenotypes, from severe combined immunodeficiency to immune dysregulation. Partial (hypomorphic) RAG deficiency (pRD) in particular, frequently leads to hyperinflammation and autoimmunity, with several underlying intrinsic and extrinsic mechanisms causing a break in tolerance centrally and peripherally during T and B cell development. However, the relative contributions of these processes to immune dysregulation remain unclear. In this review, we specifically focus on the recently described tolerance break and B cell abnormalities, as well as consequent molecular and cellular mechanisms of autoantibody production in patients with pRD.

## Introduction

A diversified repertoire of antigen receptors is generated by somatic recombination (rearrangement) of variable (V), diversity (D) and joining (J) segments (1–3). V(D)J recombination is initiated by the recombination activating gene 1 (RAG1) and RAG2, which bind to the recombination signal sequences (RSSs) flanking each gene and introduce DNA double strand breaks (DSBs) between the RSS and the adjacent *V*, *D* or *J* gene (1, 4, 5). The resulting DSBs are then processed and joined by the non-homologous end joining (NHEJ) repair pathway (1, 6–8).

B cell development in the bone marrow (BM) proceeds in a stepwise fashion and is accompanied by immunoglobulin (Ig) gene rearrangements (9). Pro-B cells undergo Ig heavy chain D to J and then V to DJ rearrangements and if successful become pre-B cells. Pre-B cells further rearrange their light chain to form IgM^+^ immature B cells (9). Absence of RAG1 or RAG2 abolishes V(D)J recombination causing a complete block in B cell development at the pro-B stage (10). Hypomorphic RAG mutations with residual recombinase activity lead to reduced V(D)J rearrangements and partial block of BM B cell development, resulting in limited Ig gene diversity and decreased but not absent peripheral B cell count (10, 11).

Due to the random nature of V(D)J recombination and the introduction of non-templated nucleotides during the joining process by NHEJ, it was suggested that up to 80% of the newly generated immature B cells are autoreactive (12, 13). Such autoreactive immature B cells are eliminated by at least three mechanisms, including clonal deletion via apoptosis, receptor editing, and functional inactivation (anergy) (12, 14). Receptor editing changes the specificity of autoreactive immature B cells by inducing a new round of light chain gene rearrangement and has been shown to be the major mechanism of central B cell tolerance (15–17). One study suggested that 25% of peripheral mature B cells in mice underwent receptor editing in the BM (18). Since receptor editing is mediated by RAG1 and RAG2, impaired central B cell tolerance could contribute to the generation of autoreactive B cells in pRD.

In humans, biallelic *RAG1* or *RAG2* pathogenic mutations lead to a broad spectrum of clinical phenotypes (1, 5, 19). While *RAG* null mutations cause T^-^B^-^ severe combined immunodeficiency (SCID) (20), pRD results in CID associated with immune dysregulation, such as Omenn syndrome (OS) (21), atypical or leaky SCID (AS or LS) (22, 23), and delayed onset combined immunodeficiency with granulomas or autoimmunity manifestations (CID-G/AI) (24, 25). pRD can also present as chronic infections with Cytomegalovirus (CMV) or Epstein-Barr virus (EBV) accompanied by γδ T cell expansion (26, 27), idiopathic CD4^+^ T cell lymphopenia (ICL) (28), common variable immunodeficiency (CVID) (29–31), and selective IgA deficiency (SIgAD) (32). Recent studies have indicated that more than half of the patients with pRD present with hyperinflammatory and/or autoimmune complications (33, 34) and produced a broad range of autoantibodies (33, 35, 36). The clinical phenotype likely depends on the nature of the defect and its quantitative and qualitative effects on V(D)J recombination, genetic modifiers and environmental factors (1, 37, 38).

In this short review, we focus on B cell abnormalities observed in patients with pRD and present an updated version of a recently published model that illustrates the molecular and cellular mechanisms underlying autoantibody production in these patients (36). This review provides additional insights into the diagnosis and treatment of RAG-deficient patients with autoimmune manifestations.

## Reduced B cell repertoire diversity in pRD

RAG1 and RAG2 initiate V(D)J recombination to generate a diverse repertoire of B cell antigen receptors. In humans, the Ig heavy chain (*IgH*) genes are located at the telomeric end of chromosome 14 and contain at least 55 functional/ORF *V_H_* genes (11 of these are not found in the reference genome), forming seven phylogenetically related subgroups (*V_H_1* to *V_H_7*) (39, 40). Downstream of the *V_H_* cluster are 23 functional *D_H_* genes, followed by 6 *J_H_* genes (39).

Recently, there has been an increase in the number of patients diagnosed with pRD and presence of peripheral B cell compartment allowed for the analysis of BCR diversity. Five studies have extensively analyzed BCR repertoire in pRD patients and mouse models (36–38, 41, 42). Lee et al. (41) examined the total B cell compartment and found reduced diversity only in OS and LS but not in CID. However, when naïve B cell compartment was assessed by Csomos et al. (38), the skewing of BCR repertoire was evident even in CID cases. Consistently, the primary repertoire of T and B cells was skewed in mouse models of pRD (42). In general, reduced BCR repertoire diversity and increased clonotypic expansion have been found in most pRD patients.

Regarding tendency for autoreactive properties in BCR, IJspeert et al. (37) analyzed the *IgH* repertoire in peripheral blood mononuclear cells and BM mononuclear cells (BMMNC) from 3 patients with similar *RAG1* mutations. While they did not find preferential usage of proximal or distal *V_H_* or *J_H_* genes, in two of the three patients they found increased frequency of *V_H_4-34*, which is known to encode intrinsically self-reactive cold agglutinin antibodies that recognize carbohydrate antigens on erythrocytes (43, 44). Lee et al. (41) studied BCR repertoire of circulating B cells in eight patients with pRD (3 CID-G/AI, 2 LS and 3 OS) and found skewed usage of *V_H_*, *D_H_* and *J_H_* genes, especially increased usage of *V_H_3-9*, *V_H_4-31*, and *V_H_3-23* in most patients. Csomos et al. (38) evaluated BCR repertoire in sorted CD38^int^, CD38^−^ and CD27^+^ B cell populations from six patients with pRD and found restricted Ig diversity with *V_H_* families being dominated by a few expanded unique clones in the “naïve” CD38^int^ population. They did not find elevated frequency of *V_H_4-34*-carrying clones in the patients. Min et al. (36) analyzed *IgH* repertoire diversity in the BM and peripheral blood of a CID-G/AI patient and a healthy control (HC) and found reduced number of CDR3 clonal peaks in the BM µ and peripheral µ and γ transcripts in the patient compared with the HC, indicating reduced Ig diversity in this patient. Intriguingly, patient BM IgG had much higher diversity than the patient peripheral IgM and IgG. In addition, 7.46% of the peripheral IgG and 1.41% of the BM IgG had identical CDR3 sequences, suggesting that these IgG^+^ cells shared the same origin (36). Along with the findings that the patient BM contained abundant CD38^high^ plasma cells (PC) and expressed high levels of IgG transcripts, these observations collectively suggest that the patient peripheral IgG^+^ B cells differentiated into IgG-secreting PC that accumulated in the BM. The analysis did not find increased CDR3 length or preferential usage of *V_H_4-34* gene in the patient B cells. Therefore, reduced BCR repertoire diversity associated with clonotypic expansion, rather than frequent usage of certain *V_H_* genes, is a common feature of pRD.

## Impaired receptor editing in pRD

Receptor editing is a RAG-dependent process and serves to change the specificity of autoreactive immature B cells (14, 45). In the BM, autoreactive immature B cells lose BCR surface expression upon self-antigen stimulation and de-differentiate back into small pre-B cells, which re-express RAG to initiate light chain gene rearrangement using available upstream *Vκ* and downstream *Jκ* genes (14). Repeated *Vκ*-*Jκ* rearrangements eventually lead to exhaustion of the recombination potential at the *Vκ* gene locus and expression of a λ light chain. Thus, distal *Jκ* usage and the proportion of Igλ^+^ B cells serves as indicators of the efficiency of receptor editing (14, 46).

Knock-in mice expressing hypomorphic RAG mutants (*Rag2^R229Q^*, *Rag1^R972Q^*, *Rag1^F971L^* or *Rag1^R972W^*) exhibit a markedly reduced proportion of Igλ-expressing splenic B cells, implicating impaired receptor editing in these mice (42, 46). Similarly, a cohort of pRD patients showed a reduced proportion of Igλ-expressing peripheral transitional B (TrB) cells (38). Moreover, reduced usage of distal *Jκ4* and *Jκ5* segments was observed in patients with OS and CID-G/AI (37, 38). Impaired receptor editing thus appears to be another common feature of pRD.

## Elevated levels of serum BAFF and increased homeostatic proliferation of B cells in pRD

B cell activating factor of the TNF-family receptor (BAFF; also known as TNFSF13B or B lymphocyte stimulator, Blys) is a membrane-bound protein that can be processed by the membrane-bound protease furin, resulting in a soluble form (47–50). It binds to three receptors, namely, BAFF receptor (BAFFR; also known as TNFRSF13C), B cell maturation antigen (BCMA; also known as TNFRSF17) and transmembrane activator and calcium modulator and cyclophilin ligand interactor (TACI; also known as TNFRSF13B) (49). BAFFR, TACI, and BCMA are expressed by B cells at different developmental and differentiation stages (48, 51). BAFFR is first expressed when immature B cells develop into TrB cells, which then receive BAFF-BAFFR dependent pro-survival signals to rescue them from premature cell death (9, 48). TACI is expressed in B cells upon activation (52) and is expressed at higher levels in marginal zone B cells (53), whereas BCMA is constitutively expressed by long-lived PC and primarily functions to support their survival through interaction with the TNF family member APRIL (a proliferation-inducing ligand) (54).

BAFF binds to BAFFR on B cells and under B lymphopenic conditions serum BAFF levels are elevated (14, 55). Assuming that a fixed amount of BAFF is produced by myeloid cells, this observation suggests a possibility that BAFF may be sequestered or degraded after binding to the BAFFR on B cells. It is also possible that chronic infections and inflammations observed in patients with pRD may result in elevated production of BAFF by myeloid cells (38, 56). pRD leads to B cell lymphopenia (33) and consistently BAFF levels have been shown to be increased in both mice and humans with pRD (36, 38, 42, 46, 56). Autoreactive TrB cells normally express low levels of BAFFR and thus have a disadvantage in BAFF-induced survival compared to non-autoreactive TrB cells (48, 57). However, in patients with pRD, serum levels of BAFF are elevated, which allows autoreactive TrB cells to survive and become mature naïve B cells.

Mature naive lymphocytes can undergo antigen independent homeostatic proliferation to sustain lymphocyte numbers in response to lymphopenic environments (58, 59). In the case of mature B cells, this process is independent of T cells (58, 60), and relies on tonic signaling through BCR, BAFF-BAFFR signaling, and recently demonstrated Notch signaling (59, 61). Transfer of resting B cells into B cell-deficient mice resulted in homeostatic expansion of a fraction of the transferred cells, which acquired an activated phenotype and differentiated into IgM-secreting cells (58, 62). In addition, in a mouse model of OS with severe B cell developmental arrest, the few remaining B cells underwent homeostatic expansion and generated a normal or even enlarged compartment of antibody-secreting cells (ASC) (46, 56). These observations in mice suggest that homeostatically expanded B cells acquire an activated phenotype and readily differentiate into ASC. In line with these observations in mice, BLIMP1^+^CD138^+^ PCs are detected in lymph node biopsies from two OS patients who are virtually devoid of circulating B cells (46). In addition, Min et al. found abundant PC in the BM of a CID-G/AI patient with few B cells in the peripheral blood (36). More recently, CD38^int^, CD38^-^ and CD27^+^ B cell subsets from patients with pRD were found to display increased activation status with higher CD69, CD80 and CD86 expression (38). These observations collectively suggest that, in patients with pRD, homeostatic proliferation allows B cells to acquire an activated phenotype and readily differentiate into PC. Additionally, reduced numbers of regulatory T cells in patients with pRD may further permit the activation and differentiation of the homeostatically expanded B cells (1).

## Enhanced B cell differentiation in pRD

Naïve B cells can be activated by antigen stimulation, toll-like receptor (TLR) ligands, cytokines and T cell-derived signals such as CD40 ligand. Activated B cells undergo proliferation, followed by differentiation into memory B or PC. CD27 has been presumed to be an exclusive marker for memory B cells, which can be further divided into CD27^+^IgD^+^ unswitched and CD27^+^IgD^-^ switched memory B cells (63). Interestingly, an earlier study identified a CD19^+^IgD^-^CD27^-^ double negative (DN) B cell population, which was phenotypically and functionally similar to CD27^+^ memory B cells, in patients with systemic lupus erythematosus (SLE) (64). Although DN B cells were also present in healthy individuals, their frequency was found to be increased in SLE and other autoimmune/autoinflammatory diseases such as mixed connective tissue disease (65) and multiple sclerosis (66), and in elderly people (67). Memory B cells can quickly differentiate into PC upon stimulation. In fact, a recent study demonstrated that DN B cells in patients with SLE were precursors of PC and upon stimulation with TLR7, IL-21, and IL-10, they efficiently differentiated into PC that secreted autoantibodies (68). It should be noted that T cell differentiation is biased toward memory/effector/helper cells in pRD and this bias may also contribute to the generation, activation and PC differentiation of these memory B-like cells (36, 38).

In some pRD patients, the distribution of B cell subsets, serum Ig levels and autoantibody production have been analyzed (25, 27, 28, 32, 36, 38, 69) (**Table 1**). These included two CID with γδ T cell expansion (γδ T^+^ CID), ten CID-G/AI, one ICL, one SIgAD, one AS, and five CID. Nineteen of the twenty patients showed increased proportions of CD27^-^IgD^-^ DN B and/or CD27^+^ memory-like B cells. Both of these subsets have the propensity to become plasma cells upon stimulation. Consistently, despite the striking reduction of peripheral B cell numbers, the majority of these patients exhibited normal or even elevated serum Ig levels (**Table 1**).

**Table 1:** B cell abnormalities and dysregulated antibody production in pRD patients.

Patient # (Gender)	Diagnosis	Gene	Variant	Recom act (% of WT)	Age	Total B (#/μl)	Naïve B (%)	Un-sw mem B (%)	Sw mem B (%)	DN B (%)	IgG (g/L)	IgA (g/L)	IgM (g/L)	IgE (IU/ml)	Autoantibody	Literatures
1 (F)	γδ T^+^ CID	RAG1	R561H R561H	2.0	11-13m	12-24↓	30.0↓	6.0	28.0↑	40.0↑	16.7↑	1.4↑	2.6↑	20	Coombs^+^	Ehl et al. (27)
2 (F)	γδ T^+^ CID	RAG1	R474C L732P	47.2 0.5	2y	63.34↓	79.3	5.3	8.1	7.3↑	6.78	0.63	0.65	NA	ANA α-DNA Coombs^+^	Asai et al. (69)
3 (M)	CID-G/AI	RAG1	W522C L541Cfs*30	41.6 1.0	14y	485↑	NA	70.7↑		NA	12.7	5.12↑	7.49↑	<5	α-AChR α-IFNα/β/ω α-IL-12p70 α-IL-22	De Ravin et al. (25), Walter et al. (35)
4 (F)	ICL	RAG1	R474C L506F	25.0 0.0	12y	Normal	20.1↓	70.6↑	8.4↓	0.9↓	7.1	0.4	0.7	9	NA	Kuijpers et al. (28)
5 (M)	SIgAD	RAG1	E455K R764H	13.73 22.42	4y	7.12↓	NA	9.5	30.6↑	NA	8.96	<0.05↓	1.22	4	NA	Kato et al. (32)
6 (M)	CID-G/AI	RAG1	c.116 + 2T>G c.116 + 2T>G	NA	8y	3.52↓	17.3↓	2.1↓	21.1↑	59.5↑	32.1↑	2.06	2.73↑	26.27	ANA pANCA PR3 Coombs^+^	Min et al. (36)
7 (M)	AS	RAG1	C328Y P619L	16 40	5m	476	91.9	2.0	0.5↓	2.4	5.3*	<0.07	0.27	NA	NA	Csomos et al. (38)
8 (M)	CID	RAG1	R474H R559S	57.8 1	2y	114↓	44.6↓	32.7↑	4.7	9.9↑	2.5↓	0.3	0.65	NA	α-IFNα	Csomos et al. (38)
9 (M)	CID-G/AI	RAG1	W522C H994R	41.6 NA	4y	70↓	36.1↓	28.7↑	11.2	13.5↑	7.53	0.40	0.31	6	Coombs^+^ α-IFNα/ω α-IL12	Csomos et al. (38)
10 (F)	CID-G/AI	RAG1	A444V A444V	1.4	5y	16↓	27.2↓	35.7↑	17.9↑	8.2↑	11.0	1.33	0.77	<30	ANA α-dsDNA APA, ACL α -β2-GPI, APS, APT Coombs^+^ α-IFNα	Csomos et al. (38)
11 (F)	CID-G/AI	RAG2	G35A G35A	22.1	12y	109↓	54.6↓	27.4↑	7.3	7.1↑	9.0	1.03	2.30	1.5	α-IFNα/ω α-IL12	Csomos et al. (38)
12 (F)	CID	RAG2	Y277fs E170G	0 14.2	13.9y	1681↑	54.5↓	29.5↑	4.3	8.2↑	4.54↓	<0.001↓	0.16	<0.1	α-IFNα	Csomos et al. (38)
13 (F)	CID-G/AI	RAG1	H612R H612R	121.6	25y	14↓	55.3	34.1↑	1.7↓	4.7	2.6	<0.07↓	0.06↓	<1	no	Csomos et al. (38)
14 (F)	CID-G/AI	RAG1	W522C R975Q	41.6 58	34y	21↓	45.5↓	34.9↑	6.6	9.3	10.47	0.76	0.98	2.3	α-IFNα/ω α-IL12	Csomos et al. (38)
15 (M)	CID	RAG1	R404W R507Q	1.7 19.4	36y	31↓	41.6↓	39.2↑	7.5	2.9	7.30*	2.58	0.025↓	<2	no	Csomos et al. (38)
16 (F)	CID	RAG1	C176F C176F	25.8	36y	10↓	23.2↓	47.0↑	11.5	4.7	13.6*	NA	NA	NA	no	Csomos et al. (38)
17 (M)	CID	RAG1	C176F C176F	25.8	38y	13↓	57.0	20.7↑	5.9	7.5	NA	NA	NA.	NA	α-IFNα α-IL12	Csomos et al. (38)
18 (F)	CID-G/AI	RAG2	F62L F62L	19.6	33y	89↓	44.4↓	39.0↑	2.7↓	8.9	6.14*↓	<0.08↓	0.17↓	<2	ANA α-IFNα/ω α-IL12	Csomos et al. (38)
19 (M)	CID-G/AI	RAG2	N173S E437K	47.5 0.9	37y	11↓	45.1↓	30.5↑	4.8↓	10.3↑	8.43*	0.22↓	0.19↓	NA	ANA Coombs^+^	Csomos et al. (38)
20 (F)	CID-G/AI	RAG2	I210T G451A	74.3 56.6	36y	18↓	20.5↓	64.4↑	9.3	1.6↓	4.67↓	9.30↑	2.34	NA	α-IFNα/ω α-IL12	Csomos et al. (38)

CID, combined immunodeficiency; CID-G/AI, CID with granulomas or autoimmunity manifestations; ICL, idiopathic CD4^+^ T cell lymphopenia; SIgAD, selective IgA deficiency; AS, atypical severe CID.

B cell subsets are shown as percentages of total B (CD19^+^) cells. Naïve B, IgD^+^CD27^-^; Un-sw mem B (Unswitched memory B), IgD^+^CD27^+^; Sw mem B (Switched memory B), IgD^-^CD27^+^; DN B, IgD^-^CD27^-^.

For patients 1-12, reference range for DN B is 3.6~5.95% (70). Reference ranges for other B cell subsets are based on the literatures shown in the table or published papers (71, 72). For patients 13-20, reference ranges were 10^th^-90^th^ percentiles calculated from relative healthy controls (38).

Recom act, recombinase activity; ANA, antinuclear Ab; α-AChR, anti-acetylcholine receptor Ab; pANCA, perinuclear anti-neutrophil cytoplasmic Ab; PR3, anti-PR3-proteinase Ab; APA, antiphospholipid Ab; ACL, anti-cardiolipin Ab; β2-GPI, β2-glycoprotein I; APS, antiphosphatidylserine Ab; APT, antiprothrombin Ab.

NA, not available; *, Ig replacement therapy; ↓, below reference range; ↑, above reference range.

Min et al. have recently reported a CID-G/AI patient caused by a c.116 + 2T>G homozygous splice site mutation in the first intron of *RAG1* (**Table 1**, Patient 6) (36). The patient had few B cells in the peripheral blood, with a remarkable 59.5% being DN B cells. Despite B cell lymphopenia, the patient BM contained a higher proportion of CD38^high^ PC and expressed elevated levels of transcripts for *PRDM1* (encoding BLIMP1) and *SDC1* (encoding CD138) compared to two HCs. An enzyme-linked immunospot (ELISPOT) assay confirmed the presence of increased number of PC in the patient BM. Interestingly, PC from the patient BM secreted higher amounts of IgM and IgG than those from the HCs. To investigate whether PCs can be generated from DN B cells, DN and naïve B cells were sorted from the peripheral blood of two patients with inborn errors of immunity (IEI) and cultured in the presence of TLR7 agonist and cytokines. Remarkably, it was found that DN B cells secreted 70-fold more IgG than did naïve B cells (36).

To explore whether the increased proportion of DN B and/or memory B cells observed in B cell lymphopenic pRD is also a general feature in patients with other IEI, Min et al. analyzed 25 immunodeficient patients with known causative genes (including *RAG1/2, PIK3CD, PIK3R1, TACI, BTK, WAS, ADA2, IKBKG, SH2D1A, CD40LG, MHCII, AICDA*) and 27 IEI patients with unknown causative genes. They found a strong association between the increased proportion of DN B and memory B (CD27^+^IgD^-^) cells and a decreased number and proportion of naive B cells in this diverse cohort (36). Notably, the proportions of DN B and memory B cells exhibited a striking increase when the count of naive B cells fell below 100 counts/µl. Therefore, the increased proportion of DN B and/or memory B cells appears to be a common characteristic in patients with IEI and B cell lymphopenia.

The pRD patients analyzed by Csomos et al. (**Table 1**) exhibited a substantial expansion of CD21^low^CD19^high^ B cell population, which is a clinically established hallmark of immune dysregulation (73–76). Moreover, CD11c^hi^CXCR5^lo^ B cell population, which is part of the CD21^low^CD19^high^ cells and resembles murine age-associated B cells (ABCs) (77), was present at a higher frequency at several B cell developmental stages in these patients (38). Consistently, the expression of T-bet, a transcription factor found in ABCs and in B cells of patients with immune dysregulation (77–79), was increased in the CD21^low^CD11c^high^ compartment (38). In addition, such CD21^low^ or CD11c^high^ B cells were found to be expanded in CVID and IEI caused by loss-of-function mutations in *CTLA4*, *LRBA*, *AICDA*, *ADA2*, *NFKB1* or gain-of-function mutations in *PIK3CD*, *STAT1*, *STAT3* and *TLR7* (80–83).

Recent studies suggest that a subset of DN B (DN2, CD27^-^IgD^-^CXCR5^-^), unswitched and switched memory-like B, CD21^low^CD19^high^ B, CD11c^high^CXCR5^low^ B and T-bet^high^CD21^low^ B cells represent a group of related B cell subsets that share the T-bet^+^CD21^low^CD11c^+^ phenotype (82). *In vitro* and *in vivo* studies indicate that T-bet is the critical transcription factor required for the generation of the CD11c^high^CD21^low^ B cells (84) and that the expansion of the T-bet^high^CD21^low^ B cells depend on BCR- and helper T cell-derived signals (81). Although the precise origin of and the relationship among these atypical B cell subsets are not fully understood, a common feature of these B cells is that they are prone to differentiate into PC upon stimulation, such as infections, and may produce autoantibodies (36, 68, 77, 82, 85, 86). These B cells may also serve as antigen-presenting cells by their high levels of CD80, CD86 and HLA-DR expression and activate autoreactive T cells (77, 82, 86), and may contribute to the pathogenesis of autoimmune/autoinflammatory diseases.

## Conclusions and perspectives

**Figure 1:**
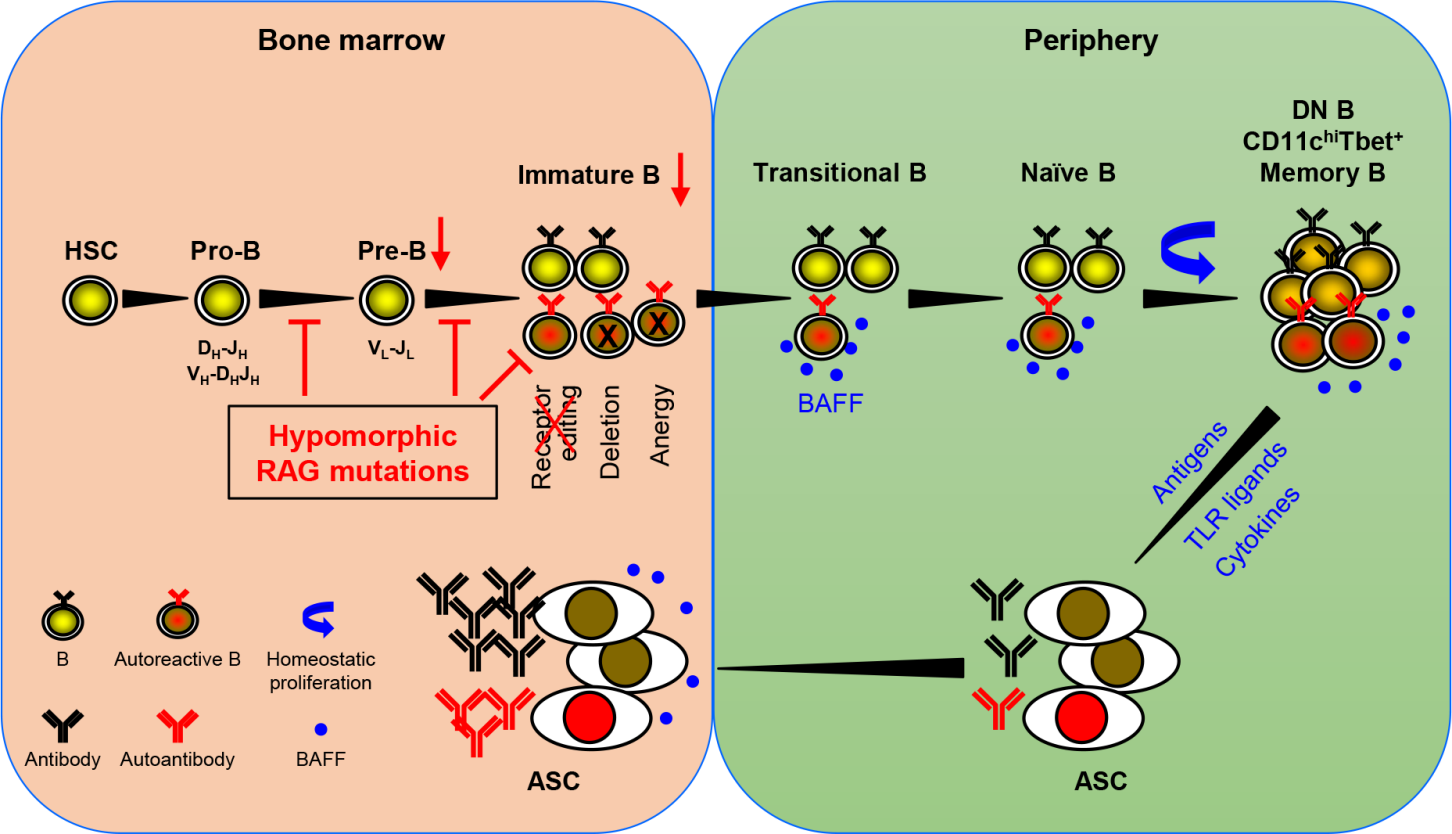
A model for autoantibody production in patients with pRD. pRD leads to limited V(D)J recombination and incomplete block in B cell development at both the pro-B to pre-B and the pre-B to immature B stages, resulting in reduced number of pre-B and immature B cells. In addition, pRD impairs receptor editing, allowing some autoreactive immature B cells to exit BM and become transitional B (TrB) cells in the periphery. Autoreactive TrB can survive in the presence of high levels of BAFF and become mature naïve B cells. These naïve B cells undergo homeostatic proliferation and differentiate into CD27^-^IgD^-^ DN, CD11c^hi^Tbet^+^ or CD27^+^ memory B cells. Note that DN B and CD11c^hi^Tbet^+^ B cells may represent two populations with similar characteristics rather than two distinct subsets. Such more differentiated B cells can efficiently become ASC upon stimulation with TLR ligands and cytokines or with foreign or self-antigens, and home to the BM. The elevated levels of serum BAFF may also promote the generation and/or survival of ASC.

Based on the available data, Min et al. have recently proposed a model for autoantibody production in patients with pRD (36). An updated version of the model is shown in **Figure 1**. *RAG* mutations impair receptor editing (37, 38, 42, 46), allowing some autoreactive immature B cells to exit BM and become TrB cells. Since serum BAFF levels are elevated in the patients with B cell lymphopenia and/or inflammation (36, 38, 56), autoreactive TrB cells can survive and become mature naive B cells. These naive B cells undergo homeostatic proliferation due to the lymphopenic environment and the presence of high levels of BAFF, resulting in the generation of DN, CD11c^high^Tbet^+^, or memory B cells (25, 27, 28, 32, 36, 38, 69). Additionally, recurrent infection and chronic inflammation in pRD may also contribute to the generation and/or accumulation of these abnormal B cell subsets. DN and CD11c^high^Tbet^+^ B cells may represent two populations with similar characteristics rather than two distinct subsets. Upon stimulation with TLR ligands and cytokines or with self- or foreign antigens, these B cells can efficiently differentiate into ASC and migrate to the BM where they secrete large amounts of antibodies, including autoantibodies.

Patients with pRD present later in life and often pose a diagnostic dilemma with clinical features combining immune deficiency and dysregulation. Recent studies have found that the immune dysregulation represents a negative prognostic factor for survival in patients with pRD, even for those who are eventually treated with hematopoietic stem cell transplantation (HSCT) (87, 88). Therefore, early diagnosis and treatment are critical to improve patient outcomes (88). Intravenous immunoglobulin (IVIG), steroids and B cell depletion using rituximab are frequently used to treat pRD patients with autoimmunity and hyperinflammation manifestations. However, these therapies often fail to control the disease (33). The model shown in **Figure 1** suggests that BAFF plays a key role in mediating the survival of autoreactive TrB cells, promoting mature B cell homeostatic expansion, and enhancing the generation of PC. Indeed, it has been reported that BAFFR blockade reduces serum levels of nucleic acid-specific autoantibodies and significantly ameliorates inflammatory tissue damage in hypomorphic RAG2-deficient mice (46). Therefore, the BAFF-neutralizing monoclonal antibody, belimumab, could be an effective treatment for pRD patients who respond poorly to the current therapeutics. This model also suggests that eliminating autoreactive ASC could be another potential approach. These therapies could be of great importance in controlling immune dysregulation and bridging the patients to HSCT, which is considered a definitive therapy.

## Author contributions

QM provided a draft of the manuscript. KC, YL, LD, ZH, XM, MY, and JW reviewed and corrected the manuscript. J-YW designed the outline and made the final corrections to the manuscript. All the authors read and approved the final manuscript.
